# Changes in the arch width and buccal corridor after fixed orthodontic treatment with Damon self-ligating system: premolar extraction vs. non-extraction

**DOI:** 10.1590/2177-6709.29.3.e2423159.oar

**Published:** 2024-07-08

**Authors:** Sarah BÜHLING, Sabrina SCHMIED, Sara ESLAMI, Silvia BRANDT, Nicolas PLEIN, Stefan KOPP, Babak SAYAHPOUR

**Affiliations:** 1 Department of Orthodontics, Johann-Wolfgang Goethe University (Frankfurt, Germany).; 2 Private practice (Taunus, Germany).; 3 Department of Prosthodontics, Johann-Wolfgang Goethe University (Frankfurt, Germany).

**Keywords:** Tooth extraction, Orthodontic appliances, fixed, Dental arch, Extração de dentes, Aparelhos ortodônticos fixos, Arcada dentária

## Abstract

**Introduction:**

Extraction vs. non-extraction is a crucial decision in orthodontic therapy.

**Objective:**

The aim of the present study was to investigate the changes in the dental arch width and buccal corridor after orthodontic treatment using extraction and non-extraction therapy with Damon self-ligating system.

**Material and Methods:**

This retrospective study consisted of 35 patients (20 female and 15 male patients with median age of 12.5 years), treated by extracting 4 or 2 premolars, and 37 patients (16 female and 21 male patients with the median age of 12.8 years), treated without premolar extraction. Both groups were treated with Damon self-ligating system. Plaster models before (T0) and after (T1) treatment were measured, and the arch width values were determined at the level of the first molars, second premolars, canines and palatal rugae. Buccal corridor width was measured using the extraoral images at T0 and T1. Paired *t*-test was used for the analysis of the normally distributed data, and Wilcoxon Mann-Whitney U test was used for the data with non-normal distribution. Values of *p*<0.05 were set as statistically significant.

**Results:**

The upper intercanine width increased significantly in both groups (*p*<0.01). In the non-extraction group, the arch width increased significantly in the maxillary second premolar and first molar region (*p*<0.01) as well as in the region of the canines (*p*=0.04), second premolars (*p*=0.01) and first molars (*p*<0.01) of the mandible. The buccal corridor decreased significantly in the non-extraction group (*p*<0.01).

**Conclusion:**

Premolar extraction in combination with Damon self-ligating system did not lead to reduction of the dental arch width in the maxilla, nor did it increase the size of the buccal corridors.

## RESUMO

**Introdução:**

Extrair ou não extrair dentes é uma decisão crucial no tratamento ortodôntico.

**Objetivo:**

O objetivo do presente estudo foi investigar as alterações na largura da arcada dentária e no corredor bucal após o tratamento ortodôntico com extração e o tratamento sem extração com o sistema autoligável Damon.

**Material e Métodos:**

Esse estudo retrospectivo consistiu de 35 pacientes (20 do sexo feminino e 15 do sexo masculino, com idade média de 12,5 anos), tratados com extração de 4 ou 2 pré-molares; e 37 pacientes (16 do sexo feminino e 21 do sexo masculino, com idade média de 12,8 anos), tratados sem extração de pré-molares. Ambos os grupos foram tratados com o sistema autoligável Damon. Os modelos de gesso antes (T0) e depois (T1) do tratamento foram medidos, e os valores de largura da arcada foram determinados ao nível dos primeiros molares, segundos pré-molares, caninos e rugas palatinas. A largura do corredor bucal foi medida usando as imagens extrabucais em T0 e T1. O teste *t* pareado foi usado para a análise dos dados com distribuição normal, e o teste U de Wilcoxon Mann-Whitney foi usado para os dados com distribuição não normal. Valores de *p* < 0,05 foram considerados estatisticamente significativos.

**Resultados:**

A distância intercaninos superiores aumentou significativamente em ambos os grupos (*p*<0,01). No grupo sem extração, a largura da arcada aumentou significativamente na região dos segundos pré-molares e primeiros molares superiores (*p*<0,01), bem como na região dos caninos (*p*=0,04), segundos pré-molares (*p*=0,01) e primeiros molares (*p*<0,01) inferiores. O corredor bucal diminuiu significativamente no grupo sem extração (*p*<0,01).

**Conclusão:**

A extração de pré-molares em combinação com o sistema autoligável Damon não levou à redução na largura da arcada dentária superior, nem aumentou o tamanho dos corredores bucais.

## INTRODUCTION

The treatment with premolar extraction, especially in borderline space deficiency cases, is one of the most controversially discussed orthodontic therapies.^1^ ^-^ ^7^ Premolar extraction is frequently deemed necessary in cases in which severe space deficiency compromises the harmonious alignment of teeth within the dental arch. This approach is essential not only to achieve proper alignment, but also to ensure long-term stability of the treatment outcome.^2^ ^,^ ^4^ ^-^ ^6^ ^,^ ^8^ However, the use of premolar extraction in orthodontic treatment has been criticized due to potential aesthetic drawbacks, such as an increase in the size of the buccal corridor.^3^ ^,^ ^8^ ^-^ ^19^ The buccal corridor describes the black triangles that are formed between the corners of the mouth and the last visible teeth on each side, when smiling.^15^ ^,^ ^20^ ^-^ ^23^ A smile that fills the lip area as much as possible, which has a narrow buccal corridor, is considered more aesthetic.^3^ ^,^ ^8^ ^-^ ^11^ ^,^ ^13^ ^,^ ^14^ ^,^ ^20^ ^,^ ^24^ ^-^ ^26^ A general correlation between the arch width and the buccal corridor has already been described, which means a decrease of the arch width can result in an increased size of the buccal corridor.^27^ ^,^ ^28^ However, the decrease of arch width in extraction cases can be avoided through the posterior expansion caused by the passive self-ligating brackets, such as Damon system (Damon Q, Ormco, Orange, CA, USA).^2^ ^-^ ^4^ Even though a recent study showed the efficacy of Damon system in avoiding arch constriction following extraction therapy,^2^ the effects of extraction therapy on the buccal corridor size and smile aesthetics using Damon system was not evaluated in this research and the evidence remains scarce.

The aesthetic result of an orthodontic treatment is one of the most essential success factors at a time when the aesthetics of the teeth, smile and face is perceived as very important in general perception.^6^ ^,^ ^29^ ^-^ ^35^ The origin of the patient’s motivation to undergo orthodontic treatment usually lies in the desire for better aesthetics, and rarely in the desire to improve the chewing function.^21^ ^,^ ^27^ ^,^ ^33^ ^,^ ^36^ ^-^ ^38^ The question arises whether orthodontic extraction therapy using self-ligating Damon system can meet the aesthetic requirements in regards to buccal corridor size. 

The aim of the present study was to compare extraction vs. non-extraction therapy using self-ligating Damon system, regarding the changes in maxillary and mandibular dental arch widths, as well as of the buccal corridor size. 

## MATERIAL AND METHODS

### STUDY DESIGN AND ETHICS

The present single-centre retrospective study was approved by the ethics committee of the medical university of the Johann-Wolfgang Goethe University (Frankfurt, Germany). (no.: 20-686). 

### SETTING

The archive of the Department of Orthodontics and Orofacial Orthopedics of the Centre for Dentistry and Oral Medicine of the Johann-Wolfgang Goethe University Frankfurt was searched to find the eligible patients for this study. 

### STUDY GROUPS

Two groups of Extraction and Non-extraction cases were included in the present study. 

### SAMPLE SIZE CALCULATION

The power calculation was based on a study by Bishara et al.^39^ In order to achieve a test power of 80% at an alpha significance level of 0.05, at least 29 patients per each group were required to detect a mean difference greater than 1.5 mm. 

### INCLUSION AND EXCLUSION CRITERIA

Only patients treated using Damon self-ligating 0.022-in slot system in both dental arches (Ormco, USA), and presenting fully erupted lower canines at T0 were included in this study. All patients presented with skeletal Class I malocclusion, neutral growth pattern, Class I or Class II molar relationship, anterior overjet of 3 to 9 mm and space deficiency of 4 to 9 mm, and were considered borderline extraction cases at T0. Patients with extraction therapy of two premolars in the maxilla or four premolars (2 in the maxilla, and 2 in the mandible) or aplasia of the relevant premolars, without partial or complete space closure, were allocated in the extraction group. 

Patients with transversal deficiency or history of treatment with expansion devices, as well as patients with dental aplasia (other than premolars and third molars), were excluded from the study.

### PATIENTS

All patients were treated with Damon self-ligating 0.022-in slot system in both dental arches (Ormco, USA), using the following archwire sequence: 0.014-in CuNiTi Damon (Ormco, USA); 0.016-in CuNiTi Damon (Ormco, USA); 0.016x0.025-in CuNiTi Damon (Ormco, USA); 0.018x0.025-in CuNiTi Damon (Ormco, USA); 0.019x0.025-in SS (Ormco, USA). 

The 35 patients in the extraction group were at the beginning of the orthodontic therapy, ranging from 7.2 to 23.2 years old (median age of 12.5 years), and were treated by extracting 4 premolars (2 in the maxilla and 2 in the mandible), or two premolars in the maxilla. The space deficiency was -6.15±3.28 mm in the maxilla and -3.37±2,88 mm in the mandible. At the end of treatment, the patients in the extraction group were between 11.7 and 27.5 years old (median age of 15.9 years).

The 37 patients in the non-extraction group (control group) were at the beginning of the orthodontic therapy, ranging from 9.1 to 17.9 years old (median age of 12.8 years), and were treated without premolar extraction. In the non-extraction group, the space deficiency was -4.46±1.31 mm in the maxilla and -2.4±1.98 mm in the mandible. At the end of treatment, the subjects in the non-extraction group were between 12.2 and 21.9 years old (median age of 16.3 years).

### INTERVENTIONS

#### Plaster models

**Figure 1:**
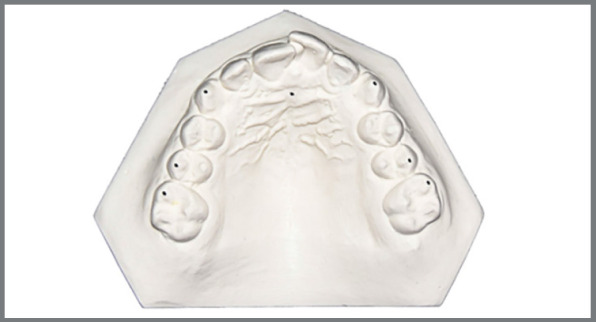
Measuring points on the maxillary plaster model.

**Figure 2:**
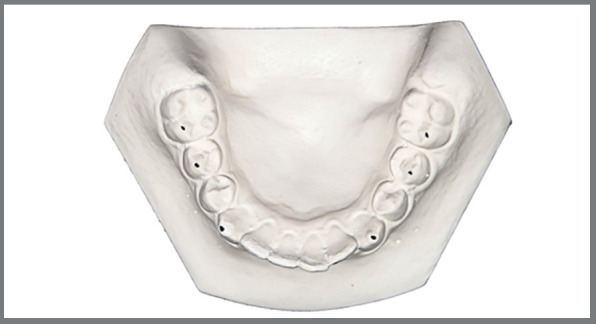
Measuring points on the mandibular plaster model.

Before (T0) and after (T1) measurements were performed on plaster models (Sheraplaster Class III, SHERA Werkstoff-Technologie GmbH & Co.KG, Lemförde/Germany), using an analogous caliper (Beerendonk, Dentaurum GmbH & Co. KG, Ispringen/Germany) (Figs. 1 and 2).

The following variables were measured (all distances were measured in millimeters):

1. Intercanine width: Distance between the cusp tips of right and left canines in upper and lower jaws.
2. Interpremolar width: Distance between the buccal cusp tips of right and left second premolars in upper and lower jaws.
3. Intermolar width: Distance between the buccal cusp tips of right and left second premolars in upper and lower jaws.
4. Arch width at the height of rugae: Distance between the outermost contours of the right and left upper teeth measured just behind the incisive papilla at right angles to the suture palatina mediana, as described by Meyer et al.^26^ ^,^ ^27^

#### Patient frontal extraoral smile images

**Figure 3:**
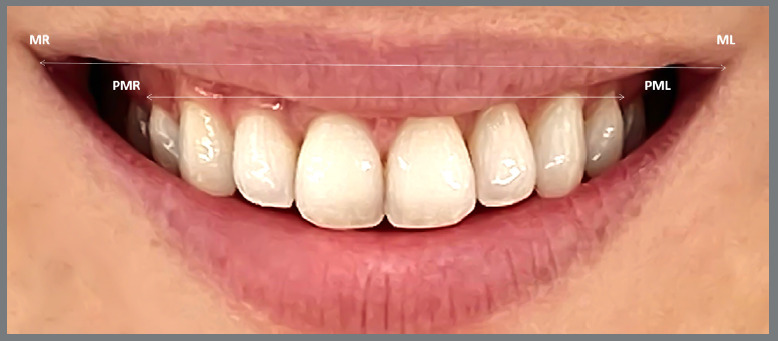
Measuring points in the smile photos. MR = inside corner of the mouth on the right side, ML = inside corner of the mouth on the left side, PMR = last visible tooth on the right side, PML = last visible tooth on the left side (OnyxCeph3™, Image Instruments GmbH, Chemnitz, Germany).

T0 and T1 buccal corridor measurements were done with the OnyxCeph analysis software (Image-Instruments, Chemnitz, Germany) (Fig. 3). 

The following parameters were measured:

1. The distance between the last visible teeth (the distance PMR to PML, being PMR = last visible tooth on the right side, PML = last visible tooth on the left side) in millimeters.
2. The smile width, measured from inside corners of the mouth (the distance MR to ML, being MR = inside corner of the mouth on the right side, ML = inside corner of the mouth on the left side) in millimeters.
3. The buccal corridor ratio, calculated according to the following formula^22^: Ratio = (distance MR to ML - distance PMR to PML): distance MR to ML*100.

#### STATISTICAL ANALYSIS

The statistical evaluation was carried out by the Institute for Biostatistics and Mathematical Modeling of the Faculty of Medicine at the J. W. Goethe University in Frankfurt using the statistics software BiAS 11.12 (Hans Ackermann BiAS for Windows).^40^ The test for normal distribution was carried out using the Kolmogoroff-Smirnoff-Lilliefors test. In order to show the differences between the groups in the development of the parameters before treatment (T0) and after treatment (T1), the two-sample *t*-test was used for normally distributed parameters. The Wilcoxon-Mann-Whitney U test was used for the parameters that were not normally distributed. To determine the effect size, Cohen’s d^41^ was evaluated in the case of the two-sample *t*-test, and Rosenthal’s R^42^ in the case of the Wilcoxon-Mann-Whitney U-test. In this connection, Cohen’s d^41^ was divided according to the following values: 0.2 = low, 0.5 = medium and 0.8 = large. Rosenthal’s R^42^ was classified according to the following values: 0.1 = low, 0.3 = medium, 0.5 = large, > 0.7 = very large. For the difference between the parameters before and after treatment within the two groups, one-sample *t*-test was used for the normally distributed, and Wilcoxon matched pairs test for the non-normally distributed data. To determine the effect size, Cohen’s d^41^ and Rosenthal’s R^42^ were evaluated. In order to test parameters for correlation, the simple linear correlation according to Pearson was used for normally distributed parameters. To determine the effect size, the correlation coefficient r was evaluated according to Evans^43^. In this context, r was considered as follows: <0.2 = poor, 0.2 - 0.4 = weak, 0.4 - 0.6 = moderate, 0.6 - 0.8 = strong and > 0.8 = optimally rated. 

All measurements were performed by a single investigator and repeated after four weeks. The mean values were calculated for reliability analysis. 

#### RESULTS

The results show the changes between time point T0 (before therapy) and T1 (after therapy).

### EXTRACTION GROUP

The arch width at the level of the canines in the maxilla increased after orthodontic therapy (T0-T1) significantly (*p*< 0.01) (Table 1). No significant change was shown in the arch widths at the level of the second premolars and at the level of the first molars in the maxilla after orthodontic treatment. The arch width at the level of the rugae in the maxilla showed no significant changes between T0 and T1. 

**Table 1:** Changes in the parameters assessed “before” and “after” treatment (T0-T1) within the extraction group (n = 35). Positive values indicate a decrease in the respective value, while negative values indicate an increase in the respective value.

Variables	T0-T1 Comparisons	SD or Min/Max	P-value	Cohen’s effect size	Evaluation
Buccal corridor ratio (%)	-0.45	3.43	0.47	0.13	1
Upper intercanine width (mm)	-2*	-5/1	< 0.01	0.55**	3
Upper interpremolar width (mm)	0.5*	-5/4.5	0.34	0.14**	1
Upper intermolar width (mm)	0.88	2.67	0.09	0.33	1
Archwidth at rugae (mm)	-0.5*	-8.5/3.5	0.16	0.2**	1
Lower intercanine width (mm)	0*	-5.5/3	0.36	0.14**	1
Lower interpremolar width (mm)	0*	-5/10	0.39	0.12**	1
Lower intermolar width (mm)	0.6	2.22	0.15	0.27	1

Statistical significance was set at *p* < 0.5. SD = standard deviation. Min = minimum. Max = Maximum.

* Use of Median and Min/Max instead of average (SD). ** Rosenthal effect size (1 = small effect size, 2 = medium effect size, 3 = large effect size).

The arch widths at the level of the canines, the second premolars and the first molars in the mandible did not show any significant changes in the extraction group between T0 and T1.

The buccal corridor ratio was not subject to any significant changes during orthodontic extraction therapy (T0-T1).

Table 1 shows the results of the changes in the parameters assessed before and after the treatment in the extraction group. Positive values ​​indicate a decrease in the respective value, while negative values indicate an increase in the respective value. 

### NON-EXTRACTION GROUP

The arch width at the level of the canines in the maxilla increased after orthodontic therapy (T0-T1) significantly (*p*< 0.01) (Table 2). The arch widths at the level of the second premolars and at the level of the first molars in the maxilla likewise increased significantly (*p* < 0.01) between T0 and T1. The arch width at the level of the rugae in the maxilla showed no significant changes after orthodontic treatment.

**Table 2:** Change in the parameters assessed “before” and “after” the treatment (T0-T1) within the non-extraction group (n = 37). Positive values indicate a decrease in the respective value, while negative values indicate an increase in the respective value.

Variables	T0-T1 Comparisons	SD or Min. /Max	*p* **-value**	Cohen’s Effect size	Evaluation
Buccal corridor ratio (%)	-0.45	3.43	0.47	0.13	1
Upper intercanine width (mm)	-2*	-5/1	< 0.01	0.55 **	3
Upper interpremolar width (mm)	0.5*	-5/4.5	0.34	0.14 **	1
Upper intermolar width (mm)	0.88	2.67	0.09	0.33	1
Archwidth at rugae (mm)	-0.5*	-8.5/3.5	0.16	0.2 **	1
Lower intercanine width (mm)	0*	-5.5/3	0.36	0.14 **	1
Lower interpremolar width (mm)	0*	-5/10	0.39	0.12 **	1
Lower intermolar width (mm)	0.6	2.22	0.15	0.27	1

* Use of Median and Min/Max instead of average (SD). ** Rosenthal effect size (1 = small effect size,

2 = medium effect size, 3 = large effect size).

In the non-extraction group, the arch widths increased significantly from T0 to T1 at the level of the canines, second premolars and first molars in the mandible (*p* = 0.04, *p* = 0.01, *p* < 0.01, respectively).

The buccal corridor decreased significantly (*p* < 0.01) from T0 to T1.

### COMPARISON OF CHANGES BEFORE (T0) AND AFTER (T1) THERAPY BETWEEN EXTRACTION AND NON-EXTRACTION GROUP

**Figure 4:**
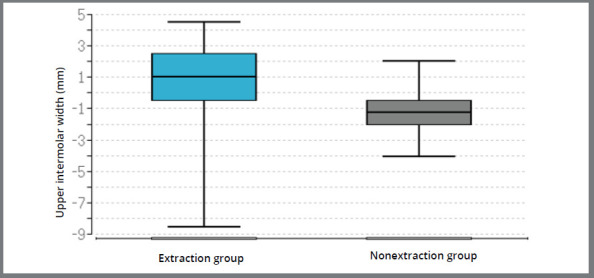
Box plots: Median and quartiles of the change in arch width at the level of the first molars in the maxilla “before” and “after” therapy in the extraction group and in the non-extraction group.

**Figure 5:**
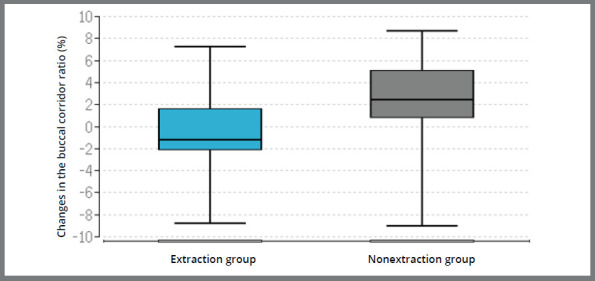
Box plots: Median and quartiles of the change in the buccal corridor ratio “before” and “after” therapy in the extraction group and in the non-extraction group.

The difference between the extraction and the non-extraction groups was not significant at the level of the maxillary and mandibular canines, but significant at the level of the maxillary second premolars (*p* < 0.01), maxillary first molars (*p* < 0.01), mandibular second premolars (*p* = 0.04) and mandibular molars (*p*< 0.01) (Table 3, Fig 4). At the level of the rugae in the maxilla, the comparison between the two groups showed no significant changes. The difference between both groups for the changes in the buccal corridor was found to be significant (*p* < 0.01) (Fig 5).

**Table 3:** Comparison of the changes “before” and “after” treatment (T0-T1) between the two groups (n = 72).

Variables	T0-T1 Comparisons (*p*-value)	Cohen’s effect size	Evaluation
Buccal corridor ratio (%)	< 0.01	0.43*	2
Upper intercanine width (mm)	0.49	0.09*	0
Upper interpremolar width (mm)	< 0.01	0.55*	3
Upper intermolar width (mm)	< 0.01	1.09	3
Archwidth at Rugae (mm)	0.88	0.02*	0
Lower intercanine width (mm)	0.59	0.07*	0
Lower interpremolar width (mm)	0.04	0.26*	1
Lower intermolar width (mm)	< 0.01	0.36*	2

Statistical significance was set at *p* < 0.5. * Rosenthal effect size (1 = small effect size, 2 = medium effect size, 3 = large effect size).

### COMPARISON OF THE EXTRACTION OF FOUR PREMOLARS OR TWO UPPER PREMOLARS IN THE EXTRACTION GROUP (TEST GROUP)

**Figure 6:**
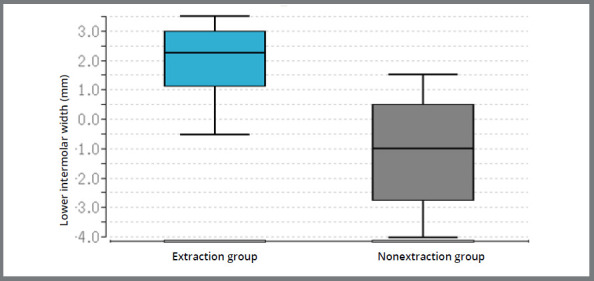
Box plots: Median and quartiles of the change in the arch width at the level of the first molars in the mandible “before” and “after” treatment in the group with extraction of 4 premolars and in the group with the extraction of 2 premolars.

The arch widths at the level of the canines in the mandible did not show any significant changes. The dental arch widths at the level of the second premolars and first molars in the mandible showed significant differences between both extraction types (*p* < 0.01) (Fig 6). The values decreased significantly after the extraction of four premolars (*p* < 0.01). After the extraction of two premolars, the values increased significantly in the area of the second premolars and first molars in the mandible (*p* < 0.01, *p* = 0.05, respectively).

No significant difference was found between the extraction methods for any of the other parameters assessed.

### CORRELATION BETWEEN DENTAL ARCH WIDTH AND BUCCAL CORRIDOR

A correlation between the change in the buccal corridor and the arch width at the level of the first molars in the maxilla was found in both groups. The results show that there was an optimal correlation between these two quantities in both groups.

## DISCUSSION

The present study evaluated the effects of extraction vs. non-extraction therapy in borderline cases using Damon self-ligating system. The results showed that extraction of premolars combined with Damon system does not affect the dental arch width or buccal corridor negatively. The common fear that premolar extraction treatment leads consequently to narrow dental arches with unaesthetic large black triangles in the buccal corridor was refuted in this study.

After premolar extraction, only the arch width at the level of the canines in the maxilla increased significantly (2mm). The arch widths at the level of the second premolars and the first molars in the maxilla and mandible, as well as the arch width at the level of the canines in the mandible, did not show any significant changes. These data show that there was no compression of the dental arch in the transversal dimension. The increased arch width at the level of the canines in the maxilla can be explained by the fact that with the distal movement of the canines after extraction, they were shifted to a wider part of the dental arch.^11^ ^,^ ^14^ ^,^ ^24^ ^,^ ^35^ ^,^ ^44^ Thus, the theory that the posterior dental arch becomes narrower as a result of mesial molar movement into a narrower part of the arch during space closure can be rejected.^11^ ^,^ ^15^ ^,^ ^16^ ^,^ ^18^ ^,^ ^24^ ^,^ ^35^ ^,^ ^44^ ^,^ ^45^ Furthermore, the arch width measurements at the level of rugae was incorporated as an additional variable in the present study, in order to mitigate potential errors arising from mesial or distal movement of landmarks. This was done to enhance the accuracy of arch width measurement and minimize any inconsistencies that may arise from the movement of reference points such as cusp tip. Contrary to the intercanine width, arch width remained stable at the height of rugae, which shows the ability of Damon system in preserving transversal dimension despite extraction therapy. 

After non-extraction therapy, the arch width significantly increased at the level of canines, second premolars and molars in both dental arches. The transversal widening of the dental arches in the non-extraction group can be explained by the harmonious shaping of the dental arch without the additional creation of space through extractions.^24^

A comparison of the buccal corridor of patients treated by extraction and non-extraction fixed appliances therapy with Damon self-ligating system shows that the buccal corridor ratio was not subject to any significant changes during orthodontic extraction therapy. This means that the fullness of the smile has remained about the same after premolar extraction therapy. 

Since the arch width at the level of the first molars and second premolars in the maxilla has remained nearly the same, the almost identical size of the buccal corridor can also be explained as an optimal correlation between the change in the size of the buccal corridor and the change in the width of the dental arch at the level of the first molars in the maxilla, which could be shown in the extraction group. In the non-extraction group, the buccal corridor decreased significantly (*p*< 0.01). The narrowing of the buccal corridor in the non-extraction group can be explained by the widening of the dental arch at the level of the first molars and second premolars in the maxilla. In this study, an optimal correlation was found between the change in the size of the buccal corridor and the change in the width of the dental arch at the level of the first molars in the maxilla in the non-extraction group. The correlation coefficient was negative in both groups, which means that if the tooth arch width is increased at the level of the first molars in the maxilla, the buccal corridor ratio (and thus the buccal corridor) becomes smaller.

The present results are partially in agreement with a study by Meyer et al,^27^ who reported an increase in the intercanine width in the extraction group and an increase in the interpremolar width in both extraction and non-extraction groups. In the non-extraction group, the arch width increased more at the level of the first molars and at the level of the rugae distal of the papilla incisiva than in the extraction group. Differences in these results compared to our study were due to the arch width at the level of the canines in our study also increased in the non-extraction group, while the arch width at the level of the rugae remained the same. The reason for this can be a greater anterior space deficiency of our patients. In addition, the arch width remained the same at the level of the premolars in the extraction group. The width of the buccal corridor after extraction therapy remained the same, whereas the buccal corridor in the non-extraction group was smaller after therapy than before the therapy. These results are exactly in line with the results of our study.

A study by Herzog et al.^24^ also examined the changes in dental arch widths in 62 Angle Class I borderline patients treated either with the extraction of four premolars or without extraction. In this study, the distance between the canines and the distance between the first molars were measured. In the extraction group, the arch width became wider at the level of the canines in the lower and upper jaws. The arch width at the level of the first molars decreased significantly in the mandible and not significantly in the maxilla. The arch widths in the non-extraction group increased in both jaws at both heights. These results are partly consistent with those of our study. In the extraction group, the distance between the canines in the maxilla also increased in our study, while the distance between the canines in the mandible remained largely the same. The distance between the first molars in both jaws also remained the same in our study, concerning the whole extraction group. In the subgroup of the extraction of four premolars, the arch width at the level of the second premolars and first molars in the mandible decreased significantly, while it increased significantly in the subgroup with the extraction of two premolars. The same results were seen in our study in the non-extraction group, where the dental arch widened at both heights. 

The differences between the therapy concepts - extraction vs. non-extraction orthodontic therapy - make the extraction decision to an individual decision, and not to a general decision between positive and negative effects.^10^

## LIMITATIONS

The demographic of the models, as well as the participants, were quite narrow in this study, what affects the generalisability of the results adversely.

Blinding of the operator was not feasible. When evaluating the models, it was evident at time point T1 whether a premolar extraction had taken place or not. Detection bias is always extant, and the results of the study were also at least partially affected by the residual growth of the participants. 

## CONCLUSION

The present study showed lack of systematic narrowing of the dental arches or an enlargement of the buccal corridor as a result of premolar extraction as a therapeutic approach for borderline cases in patients undergoing orthodontic treatment with Damon self-ligating system.

The findings of the current study indicate that the utilization of the Damon system effectively mitigates dental arch compression resulting from premolar extraction, thereby preserving the transverse dimension of the arch. This preservation of arch width contributes to maintaining the dimensions of buccal corridors. However, the transversal expansion caused by Damon system can reduce the size of the buccal corridors in borderline cases undergoing a non-extraction therapy.
